# The relationship between intelligence and reaction time varies with age: Results from three representative narrow-age age cohorts at 30, 50 and 69 years

**DOI:** 10.1016/j.intell.2017.08.001

**Published:** 2017-09

**Authors:** Geoff Der, Ian J. Deary

**Affiliations:** a MRC/CSO Social and Public Health Sciences Unit, University of Glasgow, 200 Renfield Street, Glasgow G2 3QB, Scotland; b Centre for Cognitive Ageing and Cognitive Epidemiology, University of Edinburgh, 7 George Square, Edinburgh EH8 9JZ, Scotland

## Abstract

**Background:**

Reaction time (RT) has played a prominent part in research on mental ability for over a century. Throughout this time a number of questions have been repeatedly posed: what is the relationship of RT to general mental ability, and is this the same for simple and choice RT? Does the relationship change with age? How important is RT variability compared with mean values? Here we examine these questions in three population representative cohorts.

**Methods:**

Participants were drawn from the West of Scotland Twenty-07 study, a longitudinal population based study designed to investigate socially structured health inequalities. At the fourth wave of data collection, part I of the Alice Heim 4 (AH4) test of general intelligence was administered, and reaction times were measured using a portable device. Means and standard deviations were recorded for simple and 4-choice reaction time. Full data were available for 2196 participants, comprising 714 aged 30 years, 813 aged 50, and 669 aged 69.

**Results:**

Correlations of simple RT means with AH4 scores were − 0.27, − 0.30 and − 0.32, for age 30, 50 and 69, respectively; and − 0.44, − 0.47 and − 0.53 for 4-choice RT. The underlying relationships showed evidence of non-linearity, particularly for simple RT, with stronger association at lower AH4 scores. This was more pronounced with age. RT variability was correlated with the mean at 0.57, 0.57, 0.58 for simple RT; and 0.53, 0.53, 0.47 for choice RT. Residuals from regressing the RT variability on the mean showed no association with AH4 in the case of simple RT but a weak association for choice RT which decreased with age.

**Conclusions:**

There is a strong correlation of RT means with general mental ability which increases with age. The underlying relationship is complex for SRT. RT variability shows little association with mental ability when its dependence on the mean is removed. Combining samples with disparate ages may overestimate the association.

## Highlights

• Both simple and choice reaction time are strongly correlated with IQ.
• The correlation increases with age.
• The underlying relationships are different – simple reaction time is complex and non-linear.
• Combining data from disparate ages artifactually inflates the correlation.

## 1 Introduction

Individual differences in reaction time were already being observed in the early 19th century (Brebner and Welford, 1980, Jensen, 2006) and have now played a part in research on human mental ability for well over a century. Galton undertook the first large scale observational studies of RT towards the end of the 19th century when he incorporated measurements of RT in his anthropometric laboratories (Johnson et al., 1985). From the earliest days psychologists realised that RT might be used as a measure of mental speed (which might now be termed ‘processing speed’). However, they had differing expectations of whether a purified measure of speed, such as simple reaction time (SRT), would be related to more general, higher-level mental ability. A review by Beck (1933) was inconclusive.

Reformulating the question in the framework of information theory, Hick (1952) hypothesized that RT would increase as a function of the number of choices and that this slope would be correlated with IQ but that the intercept (corresponding to SRT) would not. After some initial confirmation (Roth, 1964), subsequent work has shown both choice and simple reaction times' means to be more strongly related to mental ability test scores than the Hick slope (Deary, 2000). A review of 50 years of research on information processing speed and intelligence included RT among the measures of processing speed; it reported correlations with g in the range − 0.22 for SRT to − 0.4 for 8-choice RT and concluded that there is a trend for correlations to be higher in tasks with more choices (Sheppard & Vernon, 2008).

An assumption behind the use of the correlation coefficient is that the relationship is linear but this assumption is rarely tested. In a previous report (Der & Deary, 2003), we used data on a population based sample of 900 people aged 56 years to examine in detail the relationship of both simple and 4-choice reaction time to scores on part I of the Alice Heim 4 (Heim, 1970) test of general intelligence. The correlations were − 0.31 and − 0.49 for simple and 4-choice RT, respectively, but whereas the relationship of CRT to AH4 was approximately linear, that of SRT was complex and non-linear.

Another theme running through RT research from the very beginnings is its relationship to age. Galton's own data, later analyzed by Koga and Morant (1923), revealed the pattern still familiar today (Cerella and Hale, 1994, Dykiert et al., 2012) of initial decrease in RT through childhood followed by slower increases through adulthood accelerating in old age. However, simple and choice RT may have different patterns of ageing, with CRT declining throughout adulthood but SRT remaining relatively stable until around 60 years of age (Der & Deary, 2006).

Others have emphasised the importance of processing speed, of which RT is an indicator, in explaining cognitive ageing more generally. For example, Salthouse (1996) proposed a theory that age-related changes in fluid intelligence are mediated by changes in processing speed. Verhaeghen, building on work with Salthouse (Verhaeghen & Salthouse, 1997), summarised this and other theories of cognitive ageing and tested them against data derived from meta-analysis.

It is this area of intersection between RT, IQ and ageing that we address here. Given that SRT and CRT have different relationship to IQ and show different patterns of ageing, we hypothesized that the pattern of correlations might also differ with age. To test this hypothesis we present the correlations between RT parameters – means and standard deviations – and scores on the Alice Heim 4 test of general mental ability in three large population-representative cohorts aged around 30, 50 and 69 years.

We specifically address a number of questions with respect to SRT and CRT:

How strong is the correlation between intelligence and RT parameters?
Does the relationship between them vary with age across adulthood?
Is the underlying relationship linear?
We also examine the effect of combining the data from different ages.

## 2 Methods

Full details of the sample and measures are given elsewhere (Benzeval et al., 2008, Deary et al., 2001). A brief description follows.

### 2.1 The Sample

The data are derived from the West of Scotland Twenty-07 study: Health in the Community. This is a longitudinal population based study designed to investigate socially structured health inequalities. It comprises three age cohorts who were aged around 15, 35, and 55 when the study began in 1987, drawn as clustered random samples from the Central Clydeside Conurbation, a large urban area centred on Glasgow city. Comparison with data from the 1991 Census shows the achieved sample to be broadly representative of the population from which it was drawn (Der, 1998).

The fourth wave of data collection in 2000/2001 included the Alice Heim 4 test of general intelligence (part I) and a task measuring simple and 4-choice reaction time. Previous reports (Deary et al., 2001, Der and Deary, 2003) on the relationship between AH4 score and reaction time utilised baseline data from the oldest cohort as the other two cohorts were not administered the AH4 then.

### 2.2 The measures

Reaction times were measured using a portable device, originally designed for the UK Health and Lifestyle Survey (Cox et al., 1987). This has an LCD display screen at the top with five response buttons below it arranged in a shallow arc and labelled 1, 2, 0, 3, 4, from left to right. Our earlier article (Deary et al., 2001) includes a diagram showing the layout. For the simple reaction time test, the respondent rests the index finger of their preferred hand on the 0 button and presses it as soon as a zero appears on the screen. Eight practice trials are followed by 20 test trials. The mean and standard deviation of these trials were recorded in milliseconds. 4-choice reaction time involved the respondent resting the index and middle finger of each hand on the buttons labelled 1, 2, 3, 4 and pressing the corresponding key when one of the four digits appears in the display. There were 8 practice trials and 40 test trials. During the test trials each digit appears 10 times in a randomised order. The mean and standard deviation of reaction time are recorded separately for correct and incorrect responses as well as the number of errors. For both simple and choice reaction time the interval between a response and the display of the next digit varied randomly between 1 and 3 s. The device does not store the results of individual trials.

Period free reliabilities have been reported elsewhere (Deary & Der, 2005); test–retest correlations (Spearman's) were: SRT mean = 0.67; SRT ISD = 0.20; CRT mean = 0.92; CRT ISD = 0.73.

AH4 score is the total number of correct answers for part I of the Alice Heim 4 (Heim, 1970). This is a 65-item test, with approximately equal numbers of verbal and numerical items. The items include series completion, mental arithmetic, vocabulary, and reasoning by analogy. The time limit is 10 min. There are 12 practice items.

Part I of the test correlates 0.66 with Raven's Progressive Matrices; 0.65 with the NIIP group test 33; and the total score correlates 0.76 with the GVK and 0.63 with academic achievement in a sample of 90 schoolboys (Heim, 1970). Re-test reliability of the AH4 has been estimated at 0.919 over one month (Heim, 1970) and > 0.9 over 10 weeks (Heim & Wallace, 1950). The test has been used successfully in very large population cohorts as a valid measure of verbal and numerical reasoning (Rabbitt, Diggle, Smith, Holland, & McInnes, 2001).

### 2.3 Analysis

The methods of analysis used here for reaction time means follow those of an earlier report (Der & Deary, 2003). As before, preliminary univariate analysis confirms that the reaction times are positively skewed and that their variance decreases with increasing IQ. This suggests that methods based on ordinary least squares might not adequately represent the relationship between RT and IQ. The Box-Cox (Box & Cox, 1964) transformation is used to normalise the distributions and stabilise the variances. Then polynomial regression is used to model the relationship of RT and IQ and the results compared with those from a locally weighted regression. The predicted values from these models are transformed back to the original scale (milliseconds) and plotted for comparison. To aid interpretation of the results the bivariate distribution of RT and IQ is estimated and displayed as contours on these plots.

Because measures of reaction time variability are highly dependent on the mean, the intra-individual standard deviation, used here, was pre-processed in order to reduce the dependency on the mean before the analysis described above was applied. This was done in two ways: by calculating the coefficient of variation (CV); and by deriving residuals from a regression of the ISD on the mean.

Rather than incorporate age group and age interactions into these models, as has been done elsewhere, we performed the modelling separately by age cohort. Previous work (Der & Deary, 2006) had shown that the ISD might be best fit as a non-linear function of the mean, plus, in the case of choice RT ISD, a non-linear function of the number of errors. Consequently, to model these relationships we employed polynomial regression with backwards elimination from the highest powers (Fox, 1997). For SRT backwards elimination starting from the fifth power resulted in a cubic function for the older two cohorts and a quartic for the 1970s cohort. For CRT the starting points were the fourth power of the mean and cubic in number of errors. This yielded models that were linear in the number of errors and in the mean for the older two cohorts, but quadratic in the mean for the youngest, 1970s, cohort.

To assess the potential impact of attrition in the study we repeat the main results using inverse probability weights.

All analyses and pre-processing were carried out separately for each age cohort.

SAS version 9.3 was used throughout.

## 3 Results

A total of 2661 respondents took part in the fourth wave of the Twenty-07 study. There were missing data for the AH4 in 334 cases: in 100 of these the respondent refused. In 155 cases the interviewer did not administer the AH4, for a variety of reasons mostly because the conditions were not deemed appropriate for a fair assessment. In the remaining 79 cases no reason was given. There were missing data for one or more of the RT measures in a further 128 cases. Two respondents were excluded for excessive errors and one where the tests were administered in error. This left an analysis sample of 2196: 714 aged 30, 813 aged 50 and 669 aged 69.

Table 1 gives descriptive statistics for the reaction time measures and AH4 scores by age cohort. With respect to the ages of the cohorts, it is worth noting the narrow spread of ages around the cohort mean as indicated by standard deviations of 1.0 and 1.3. As expected, reaction time means increase with age and AH4 score decreases. The raw reaction time intra-individual variability (ISD) also increases with age for both simple and choice reaction measures. The coefficient of variation is not clearly related to age for either simple or choice RT. Nonetheless, with the sample size here cohort differences are all statistically significant at p < 0.001 for all measures except the ISD residuals. The derivation of the ISD residuals was described above but as these were calculated separately for each age cohort they have a mean of zero and no association with age.

**Table 1:** Descriptive statistics.

		Cohort		
		1970s	1950s	1930s
Age	Mean	30	50	69
	SD	1.3	1.3	1.0
AH4 score	Mean	39	36	28
	SD	10	12	11
	Skewness	− 0.2	− 0.1	0.2
Simple RT mean	Mean	290	318	354
	SD	74	97	112
	Skewness	2.0	2.0	1.6
Choice RT mean	Mean	539	623	729
	SD	72	93	105
	Skewness	0.7	1.1	0.8
Simple RT ISD	Mean	72	83	98
	SD	48	59	63
	Skewness	2.6	2.2	1.5
Choice RT ISD	Mean	100	121	140
	SD	29	35	37
	Skewness	1.2	1.4	0.9
SRT CV	Mean	0.24	0.25	0.27
	SD	0.12	0.15	0.14
	Skewness	2.2	2.5	1.7
CRT CV	Mean	0.18	0.19	0.19
	SD	0.04	0.05	0.04
	Skewness	1.2	1.0	0.8
SRT ISD residuals	Mean	− 0	0	0
	SD	39	48	51
	Skewness	2.3	2.1	1.4
CRT ISD residuals	Mean	0	0	0
	SD	24	30	32
	Skewness	1.1	1.0	0.7
All	N	714	813	669

All RT measures are in milliseconds.

All reaction time measures are positively skewed, particularly those for simple reaction time. Whereas this is well established for the mean and ISD, it is notable that similar values are evident for the measures of ISD which control for the mean - the coefficient of variation and the residuals from a regression on the mean. Controlling for the mean has barely affected the skewness of their distributions. As we have argued previously, the levels of skew together with heteroscedasticity in the relationship to IQ have the potential to bias the estimation of that relationship.

Table 2 shows the correlations between the RT variability measures and their corresponding means by age cohort. There is a substantial correlation between the ‘raw’ ISD and the mean both for simple and choice RT. The coefficient of variation is only partially successful at removing this association with the mean. For simple RT the correlation is much lower but by no means negligible. For CRT there are mixed results; some correlation remains for the youngest cohort, zero correlation for the 1950s cohort, and a small negative correlation for the 1930s cohort, albeit this has only marginal significance. For the residuals there is zero correlation in all cases, as expected.

**Table 2:** Correlation of RT ISD measures with corresponding RT mean.

	Cohort					
	1970s		1950s		1930s	
	r	p	r	p	r	p
SRT ISD	0.57	< 0.001	0.57	< 0.001	0.58	< 0.001
SRT CV	0.22	< 0.001	0.17	< 0.001	0.14	< 0.001
SRT residuals	− 0.00	–	0.00	–	0.00	–
CRT ISD	0.53	< 0.001	0.53	< 0.001	0.47	< 0.001
CRT CV	0.10	0.008	0.01	0.837	− 0.08	0.048
CRT residuals	0.00	–	0.00	–	0.00	–

Table 3 shows the correlations between the AH4 scores and reaction time measures for the cohorts individually and combined. We consider first the results for the individual cohorts. For CRT mean the correlation increases with age from − 0.44 at age 30, to − 0.47 at 50, and − 0.53 at 69. For SRT mean the correlation increases but less markedly so from − 0.27 at age 30 to − 0.30 at 50, and − 0.32 at 69. Previously-reported correlations for the oldest cohort when they were aged around 56 (Deary et al., 2001) were − 0.31 and, for SRT and − 0.49 for CRT.

**Table 3:** Correlation of RT measure with AH4 score by individual cohort, cohorts combined and cohort combined, partialled for age.

	Cohort				
	1970s	1950s	1930s	Combined	Combined age partialled
SRT mean	− 0.27⁎⁎⁎	− 0.30⁎⁎⁎	− 0.32⁎⁎⁎	− 0.36⁎⁎⁎	− 0.30⁎⁎⁎
CRT mean	− 0.44⁎⁎⁎	− 0.47⁎⁎⁎	− 0.53⁎⁎⁎	− 0.58⁎⁎⁎	− 0.49⁎⁎⁎
SRT ISD	− 0.21⁎⁎⁎	− 0.21⁎⁎⁎	− 0.24⁎⁎⁎	− 0.27⁎⁎⁎	− 0.23⁎⁎⁎
CRT ISD	− 0.34⁎⁎⁎	− 0.31⁎⁎⁎	− 0.26⁎⁎⁎	− 0.41⁎⁎⁎	− 0.30⁎⁎⁎
SRT CV	− 0.14⁎⁎⁎	− 0.10⁎⁎	− 0.11⁎⁎	− 0.14⁎⁎⁎	− 0.12⁎⁎⁎
CRT CV	− 0.17⁎⁎⁎	− 0.08⁎	0.04	− 0.09⁎⁎⁎	− 0.06⁎⁎
SRT residuals	− 0.06	− 0.04	− 0.05	− 0.04⁎	− 0.05⁎
CRT residuals	− 0.12⁎⁎	− 0.07	− 0.00	− 0.05⁎	− 0.06⁎⁎

⁎ p < 0.05.

⁎⁎ p < 0.01.

⁎⁎⁎ p < 0.001.

For CRT ISD the correlation appears to be decreasing, whereas for SRT ISD there is no clear pattern. Previously reported values for the oldest cohort were − 0.26 for both SRT and CRT ISD.

For the coefficient of variation the pattern of correlations with AH4 appear to follow that with the mean; the SRT CV correlations are substantially lower than the unadjusted ISD, but with a small correlation remaining; the CRT CV correlations are also substantially lower than the unadjusted ISD but, as with the mean, decrease with age effectively to zero in the oldest cohort; the small positive correlation for that cohort is non-significant. The SRT residuals have correlations which are lower again, just under half those of the CV and about a quarter of the raw ISD correlations. The CRT residuals' correlation with AH4 decreases with age reaching zero in the oldest cohort.

When the cohorts are combined somewhat paradoxical results are obtained: for the mean and ISD of both SRT and CRT the values lie outside the range spanned by the individual cohorts and are larger in absolute terms. So, for example, the correlation with SRT mean is − 0.36 when the cohorts are combined, whereas the values for the individual cohorts range from − 0.27 to − 0.32. For CRT mean the correlation is − 0.58 in the combined cohorts but ranges from − 0.44 to − 0.53 in the individual cohorts. The results for SRT and CRT ISD exhibit the same pattern. These seemingly paradoxical results are examples of the reversal paradox a phenomenon that also encompasses Simpson's paradox and Lord's paradox (Tu, Gunnell, & Gilthorpe, 2008). They arise because RT means, ISDs and AH4 scores are all correlated with age as is evident in Table 1. The purified residuals are uncorrelated with age by design so the combined correlation with AH4 scores is in the middle of the range of separate cohort results as might be expected. The coefficient of variation is somewhat intermediate.

When age is partialled out the results are in the mid range of the cohort specific estimates. Partialling out cohort, instead of age, gives virtually identical results (results not shown) as the correlation between age and cohort is > 0.99 due to the narrow age range within cohorts.

**Fig. 1:**
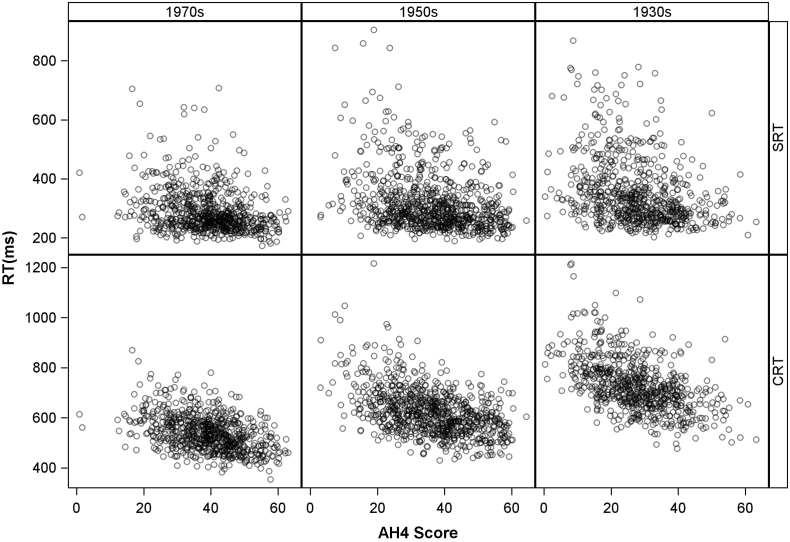
Scatter plots of RT mean by AH4 scores - values jittered.

We now turn to the results that examine in detail the relationships underlying the correlations. Fig. 1 shows scatterplots of both reaction time means against AH4 score. A negative association is evident for choice reaction time, but less so for simple reaction time. For simple reaction time, there appears to be a floor effect just above 200 milliseconds. There is a strong suggestion of decreasing variance with increasing AH4 score. Thus the data exhibit both positive skew and heteroscedasticity.

**Fig. 2:**
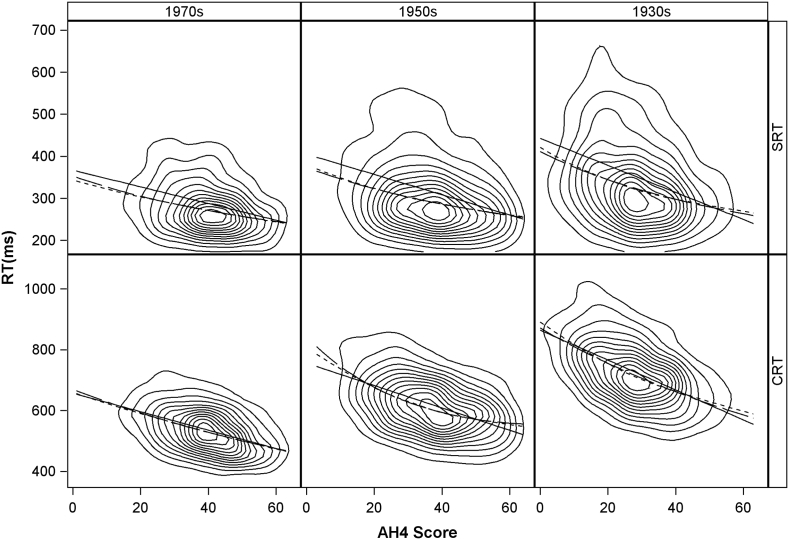
Predicted values from regression of RT means on AH4 scores overlaid on contours of the estimated bivariate density. Solid line = OLS regression; long dash = polynomial Box-Cox regression; short dash = Loess Box-Cox regression

As in our previous report, we used the Box-Cox procedure (1964) to derive an optimal transformation for the data. The transformed reaction times were then modelled with a polynomial regression on AH4 score using backward elimination from the 4th power. Finally, as a check that the parametric form does not unduly influence the fit, especially at the extremes, we fitted a locally weighted (loess) regression line with the equivalent degrees of freedom of a quadratic regression (Hastie & Tibshirani, 1990). The predicted values from each of the regressions are displayed in Fig. 2 overlaid on contour plots of the estimated bivariate distributions.

For simple reaction time, the heteroscedasticity is the most striking feature of the relationship (Fig. 2). That is to say, the variation between individuals in their SRT mean is much greater at lower IQ levels. We noted this pattern in our previous report, but here we can also see that this pattern is accentuated with increasing age. In comparison, the peak of the bivariate distribution, contained within the innermost contours, has a less marked relationship to age. The ordinary regression line lies some way above the peak and, hence, is a biased summary of the bivariate relationship. This bias is not surprising given skewness and non-constant variance. The regression of the transformed reaction times is an improvement showing an association that is steeper at lower IQ levels. The locally weighted regression is virtually identical.

For choice reaction time, the contours form a pattern much closer to the series of concentric ellipses typical of a bivariate normal distribution (Fig. 2). The whole distribution is shifted up and to the left with age, corresponding to a simultaneous slowing of CRT and decline in AH4 scores. There is a tendency for inter-individual variability at lower AH4 scores to increase with older age, but this is much less marked than with SRT. The ordinary least squares regression of the original data is a much better summary of the relationship. It bisects the inner contour in the 1970s cohort and is only slightly above for the older two. Polynomial regression using the transformed data results in a quadratic relationship in the 1950s cohort but is closely aligned with the ordinary regression in the other two cohorts. The polynomial and locally weighted regressions are again in close agreement.

Detailed results for the RT ISD, before and after correcting for the mean, are shown in Appendix A.

Correlations between RT measures and AH4 scores weighted for attrition are shown in Appendix B. There are few differences of note, the possible exceptions being stronger correlations for SRT and CRT means in the oldest (1930s) cohort and weaker correlations for CRT ISD and CRT CV in the youngest (1970s) cohort.

## 4 Discussion

In these large, population-representative cohorts reaction time means are strongly correlated with IQ. This is true for both simple and 4-choice RT although the underlying relationships are different. The correlations increase somewhat with age. However, combining groups of disparate ages can exaggerate the correlation markedly.

Reaction time variability is also correlated with IQ but this largely reflects the interdependence of the variability and the mean. Once the dependence on the mean is accounted for the relationship to IQ is weak and inconsistent.

The results here for the 1950s cohort at age 50 are in close agreement with those for the 1930s cohort reported previously when they were aged 56. The correlations then of AH4 scores with simple and 4-choice reaction time means were − 0.31 and − 0.49, respectively, compared with − 0.30 and − 0.047 reported here. Even these very small differences barely detract from the agreement given the trend towards increasing correlation with age which is discussed below.

The correlations reported here are somewhat larger than typically reported elsewhere, for example in Jensen's, 1987 review (Jensen, 1987). A more recent review (Sheppard & Vernon, 2008) reported a summary correlation between ‘overall’ RT and *g* of − 0.26 based on 112 samples. The individual correlations ranged from − 0.65 to 0.06 (data made available by the authors). Restricting the results to the 93 adult samples, gives a summary value of − 0.29 (SD 0.16; range − 0.61 to 0.06). Thus, our results are well within the range of previously reported results. It is also worth comparing our results for CRT with the meta-analytic summary correlation of 0.53 between ‘speed’ and ‘reasoning’ obtained by Verhaeghen (2013) even though his measure of speed included both RT tasks and other speeded tests. An important point which must be born in mind when considering the correlations is that the AH4 is a time limited task and therefore incorporates an element of speed.

A strength of our study is the large sample sizes. The 93 adult samples in the review of Sheppard and Vernon included our previous report for the 1930s cohort (N = 900) and two military samples with Ns of 397 and 303; all other Ns were < 200 and the median sample size was 73. Variability in the estimates from previous studies would, therefore, be expected due to sampling error.

There are also reasons why some studies might have under-estimated the correlation. The numerous studies based on student populations could give attenuated estimates because of their restricted ability range. Furthermore, correlations between psychometric tests and information processing measures tend to be higher in lower ability samples than in high ability samples (Deary et al., 1996, Detterman and Daniel, 1989, Legree et al., 1996).

Another source of variability arises from the reaction time paradigm employed. Many studies use the paradigm devised by Jensen and Munro (Jensen & Munro, 1979) which is designed to separate decision time and movement time. The device and protocol used here do not involve any movement prior to the pressing of a button and, arguably, comes closer to a pure measure of reaction time.

The results here suggest that the age composition of samples may play a complex role in determining the strength of the correlation. On the one hand, the correlation increases somewhat with age but samples that combine people of disparate ages may also overestimate the correlation.

The general trend towards increasing correlation with age, which might support the dedifferentiation hypothesis (Balinsky, 1941), is more evident for CRT than SRT. A difference between the two would be expected given their different relationships to age. In the HALS study, which used the same RT device and procedure in a large, all-age, sample of adults (Der & Deary, 2006), the CRT mean increased continuously throughout the adult age range, whereas SRT mean was flat until around 60 years of age after which it started to increase.

Considering the details of the relationships underlying these correlations, the results for the 1950s cohort presented, here at age 50, are also very similar to the previous report of the 1930s cohort when they were aged 56. For SRT these are: the heteroscedasticity, with greater inter-individual variation in RT at lower AH4 scores and the floor effect at or around 200 ms. The new finding from these results is that the heteroscedasticity is, itself, related to age and increases with age. Thus the differential correlation in high and low ability groups noted in the studies cited above might also depend on age. However, this effect is more evident for SRT than it is for CRT.

The correlation between CRT and AH4 increases with age, but there is little evidence of an increase in spread at lower ability levels. There is some departure from linearity but it is slight. The differential correlation in high and low ability groups also implies the existence of a non-linear relationship when the whole ability range is taken together but this is only clearly born out for SRT results. One aspect of SRT that plays a part in these differences is the floor effect which also varies with age and is more apparent in the younger cohorts.

When data from the three cohorts are combined seemingly paradoxical results are obtained for correlations between AH4 scores and RT means and ISDs, the values are all outside the range of values obtained for the cohorts individually. This is due to the fact that RT means ISDs and AH4 scores are all associated with age. Partialling on age or cohort gives results that are in the mid range of the cohort specific results as might be expected. Similar effects would be expected in an all-age sample covering the same age range. Hofer and Sliwinski (2001) warned against the possibility of artefacts like this in cognitive ageing research with a number of hypothetical scenarios. The results here reinforce the message with a striking real world example.

## 5 Conclusions

In these large population-based samples of young-, middle-ages, and older- adults, both simple and 4-choice reaction time means show strong negative associations with general intelligence. The size and nature of the samples, together with the relative simplicity of the reaction time task, afford some authority to these estimates.

For both simple and 4-choice, the correlation increases with age. Simple reaction time presents a more complex picture which is dependent both on age and level of ability. Reaction time variability, as measured by the intra-individual standard deviation, is highly correlated with the mean. The coefficient of variation fails to fully account for this dependency and so gives results that are hard to interpret. ‘Purified’ residuals give a clearer picture; for simple reaction time they show no association and, for choice reaction time, a weak association that decreases with age.

Researchers concerned with the intersection of cognition, processing speed and ageing, such as in information processing theories of cognitive decline, need to be aware of the complexities of the underlying relationships and of the artefacts of combining groups of disparate ages.

## Figures and Tables

**Fig. 3:**
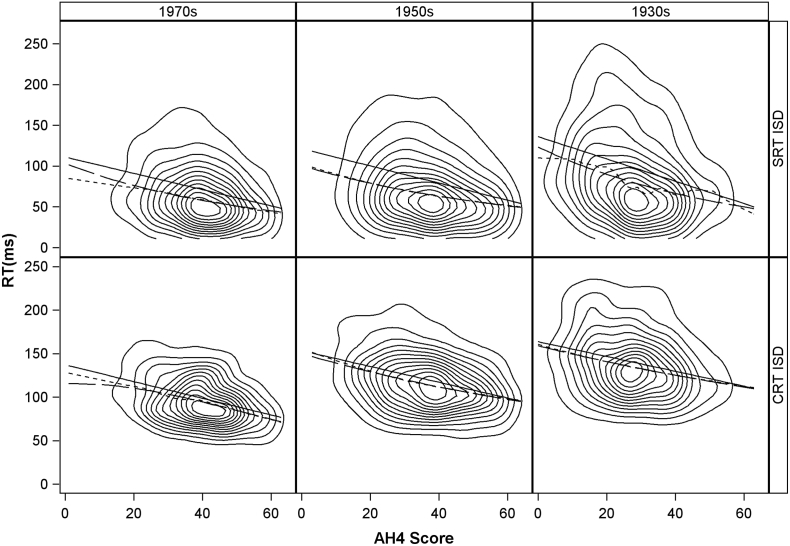
Predicted values from regression of RT ISD on AH4 scores overlaid on contours of the estimated bivariate density. Solid line = OLS regression; long dash = polynomial Box-Cox regression; short dash = Loess Box-Cox regression

**Fig. 4:**
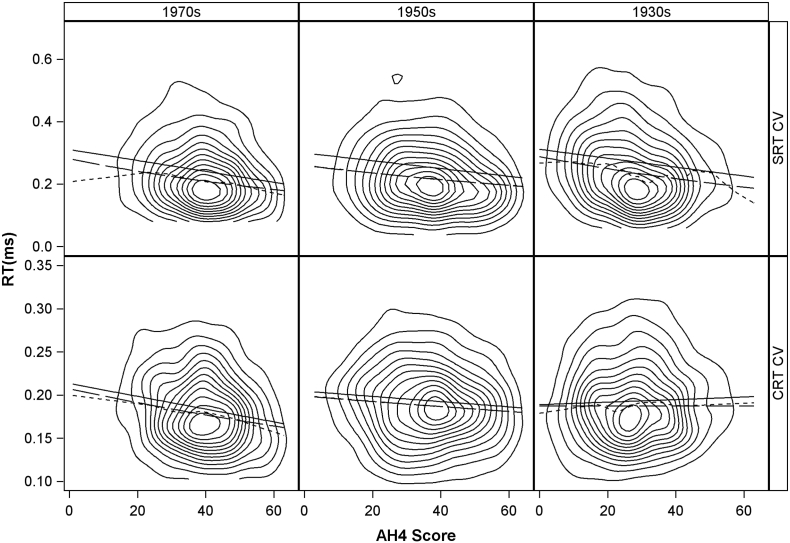
Predicted values from regression of RT CV on AH4 scores overlaid on contours of the estimated bivariate density. Solid line = OLS regression; long dash = polynomial Box-Cox regression; short dash = Loess Box-Cox regression

**Fig. 5:**
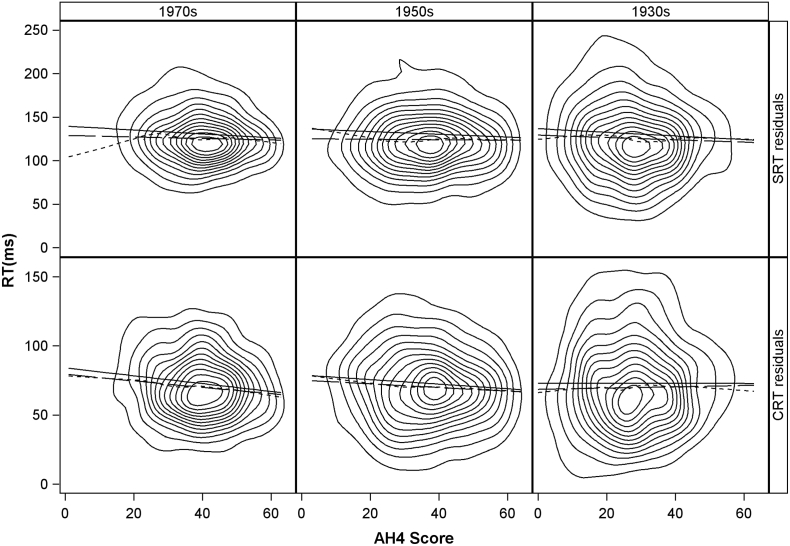
Predicted values from regression of RT ISD residuals on AH4 scores overlaid on contours of the estimated bivariate density. Solid line = OLS regression; long dash = polynomial Box-Cox regression; short dash = Loess Box-Cox regression

**Table:** 

	Cohort		
	1970s	1950s	1930s
SRT mean	− 0.28⁎⁎⁎	− 0.31⁎⁎⁎	− 0.37⁎⁎⁎
CRT mean	− 0.45⁎⁎⁎	− 0.47⁎⁎⁎	− 0.55⁎⁎⁎
SRT ISD	− 0.22⁎⁎⁎	− 0.21⁎⁎⁎	− 0.25⁎⁎⁎
CRT ISD	− 0.31⁎⁎⁎	− 0.30⁎⁎⁎	− 0.25⁎⁎⁎
SRT CV	− 0.14⁎⁎⁎	− 0.08⁎	− 0.09⁎
CRT CV	− 0.14⁎⁎⁎	− 0.07	0.05
SRT residuals	− 0.05	− 0.02	− 0.01
CRT residuals	− 0.09⁎	− 0.06	0.02

** p < 0.01.

⁎ p < 0.05.

⁎⁎⁎ p < 0.001.

## Appendix A Detailed results and discussion of the relationship between RT variability and IQ

Fig. 3 gives the results for the analysis of intra-individual standard deviations in the same format as given for the RT means in Fig. 2. The pattern for SRT closely mirrors that for the SRT mean as might be expected from the strong correlation between the mean and ISD shown in Table 2. The results for CRT ISD are less similar to those for the mean. Again this is to be expected given the weaker correlations of the CRT ISD both with its mean and with the AH4 score.

Fig. 4 presents the results for the coefficient of variation of SRT and CRT. As noted earlier, the SRT CV retains a moderate correlation with the mean and a reduced but still significant correlation with the AH4. Some of the heteroscedasticity evident for the SRT mean can be seen with the SRT CV, although it is much less marked. The ordinary regression lines are similar to the results of polynomial regression on the transformed values in that the latter yield linear relationships with similar slopes but a lower intercept. The locally weighted regressions show some differences, but these do not appear systematic. For CRT CV, the contours are closer to the circular pattern typical of a null relationship and the three regression lines are in close agreement for each cohort, but the relationship weakens with age.

Fig. 5 presents the results for the RT ISD residuals. For the SRT ISD residuals there is very little sign of a remaining relationship to AH4. Although the ordinary regressions appear to show a weak relationship, none of these or the regressions of the transformed variable are significant (results not shown). For CRT ISD residuals there is a weak but significant relationship to AH4 in the youngest, 1970s, cohort only.

### Discussion

Reaction time intra-individual variability has attracted attention as a potentially useful measure of cognition. Some studies show it to be more strongly related to IQ than RT mean, but others show the opposite. Again the issue is confounded by differences between samples, particularly in their age. Perhaps more importantly, ‘raw’ measures of intra-individual variability such as the within person standard deviation of RT across trials in an RT task are typically correlated with the mean, as is the case here with values around 0.57 for SRT and 0.47 to 0.53 for CRT. Thus, in order to understand the specific role of IIV, it is necessary to remove the dependence on the mean. Here we have examined the results of two common methods used to do this: the coefficient of variation and residuals from a regression on the mean and other relevant parameters.

The coefficient of variation has the advantage of being a simple measure which is easily derived from summary statistics. However, the results here suggest that it is not fully effective at removing the dependence on the mean; for SRT the correlation with the mean was reduced but by no means eliminated, whereas for CRT its effectiveness was unclear – a significant correlation in the 1970s cohort, but zero and possibly a small negative correlation in the 1950s and 1970s cohorts, respectively. These results were reflected in the correlations between the CV and the AH4; for SRT they are reduced but not eliminated; for CRT the correlation is reduced by a half in the 1970s cohort, by two thirds in the 1950s cohort and eliminated in the 1930s cohort.

Where there are significant correlations between the CV and AH4 these are larger than would be expected simply by virtue of the remaining correlation with the mean. A rough approximation of the correlation due to the residual correlation with the mean could be made by multiplying the two correlation coefficients. For example, in the 1970s cohort SRT CV this would be − 0.06 (0.22 × − 0.27) and for the other two cohorts − 0.05 whereas the correlations of the SRT CV with AH4 are around twice these values. However, it is difficult to interpret the results given that the approximation ignores all the complexity of the underlying relationships already illustrated. In short, because the SRT CV is related to the mean it is difficult to interpret any association with AH4.

The correlation of CRT CV with AH4 depends on age. It follows the same decreasing pattern as the correlation with the mean but it not determined by that. For example, in the 1970s cohort the correlation of − 0.17 is larger than the correlation with the mean and nearly four times the correlation with AH4 that would be due to the remaining correlation with the mean. Since the correlation with the mean was effectively zero in the 1950s cohort, the correlation with AH4 score is clearly not due to any remaining correlation with the mean. In the 1930s cohort the correlation again has the opposite sign, but is not even marginally significant. Moreover, the detailed relationship illustrated in Fig. 3 gives very little support for a non-zero correlation.

The residuals obtained by regressing the RT ISD on the RT mean have, by definition, a zero correlation with the mean and, in this respect, a distinct advantage over the coefficient of variation. For SRT there is little evidence that they are related to AH4 scores, whereas for CRT there is a relationship in the youngest, 1970s, cohort which decreases to zero in the oldest. This result is somewhat at odds with the suggestion from elsewhere in the literature that RT variability is associated with cognitive decline, which would imply an increasing correlation with age. If RT variability is intrinsically associated with cognitive decline, rather than through the RT mean, then it is likely to be due to some characteristic other than general intelligence. One possibility raised by the results here, is that it might be an artefact of combining data from people of different ages. The artefact demonstrated here would be greatest where the underlying relationships to age are strongest, in particular among older aged groups.
